# Genome-Wide Transcriptome Analysis of Cadmium Stress in Rice

**DOI:** 10.1155/2016/9739505

**Published:** 2016-02-29

**Authors:** Youko Oono, Takayuki Yazawa, Hiroyuki Kanamori, Harumi Sasaki, Satomi Mori, Hirokazu Handa, Takashi Matsumoto

**Affiliations:** Agrogenomics Research Center, National Institute of Agrobiological Sciences, Tsukuba, Ibaraki 305-8602, Japan

## Abstract

Rice growth is severely affected by toxic concentrations of the nonessential heavy metal cadmium (Cd). To elucidate the molecular basis of the response to Cd stress, we performed mRNA sequencing of rice following our previous study on exposure to high concentrations of Cd (Oono et al., 2014). In this study, rice plants were hydroponically treated with low concentrations of Cd and approximately 211 million sequence reads were mapped onto the IRGSP-1.0 reference rice genome sequence. Many genes, including some identified under high Cd concentration exposure in our previous study, were found to be responsive to low Cd exposure, with an average of about 11,000 transcripts from each condition. However, genes expressed constitutively across the developmental course responded only slightly to low Cd concentrations, in contrast to their clear response to high Cd concentration, which causes fatal damage to rice seedlings according to phenotypic changes. The expression of metal ion transporter genes tended to correlate with Cd concentration, suggesting the potential of the RNA-Seq strategy to reveal novel Cd-responsive transporters by analyzing gene expression under different Cd concentrations. This study could help to develop novel strategies for improving tolerance to Cd exposure in rice and other cereal crops.

## 1. Introduction

Cadmium (Cd) is a widespread heavy metal pollutant that is highly toxic to living cells. Accumulation of the nonessential metal Cd in plants is a major agricultural problem. Specifically, Cd is absorbed by the roots from the soil and transported to the shoot, negatively affecting nutrient uptake and homeostasis in plants, even in very small amounts. Many agricultural soils have become contaminated with Cd through the use of phosphate fertilizers, sludge, and irrigation water containing Cd. Cd exposure inhibits root and shoot growth and ultimately reduces yield. Furthermore, Cd accumulation in the edible parts of plants such as seed grains places humans at a risk because of its highly toxic effects on human health. Reducing the Cd concentration in plants below the maximum level indicated by the Codex Alimentarius Commission of FAO/WHO [1] is necessary to avoid negative impacts on human health. Thus, it is important to study the mechanisms of plant responses and defenses to Cd exposure to overcome this problem.

Cd causes oxidative stress and generates reactive oxygen species, which can cause damage in various ways such as reacting with DNA causing mutation, modifying protein side chains, and destroying phospholipids [2]. Various biochemical and physiological processes associated with defense systems are active in plants under Cd exposure. Many genes such as glutathione S-transferase (GST) for detoxification and cysteine-rich metallothioneins (MT) for defense against Cd toxicity respond to Cd stress in plants and might confer Cd tolerance in rice. Transporters with heavy metal binding domains are key factors for root uptake of Cd from soil and efflux pumping of Cd at the plasma membrane; however, the manner in which these genes respond to low Cd concentrations has not been well investigated in rice.

In a previous study, we investigated the gene expression of rice plants (*Oryza sativa* L. cv. Nipponbare) under a high Cd concentration using the RNA-Seq platform. A clear and detailed view of the transcriptomic changes triggered by Cd exposure is important to understand the gene expression network of the basal response to Cd stress. This could not be obtained from past studies using the microarray platform, but RNA-Seq can accurately quantify gene expression levels over a broad dynamic range with high resolution and sensitivity [3]. We found that drought stress signaling pathways were activated under Cd exposure through the responses of many drought-related genes [4]. Thus, the recently elucidated scaffolding mechanisms for Cd signaling pathways are complex but of great importance. In this study, we performed rice transcriptome analysis under different low Cd concentrations using the RNA-Seq platform to deepen our understanding of Cd responses.

## 2. Materials and Methods

### 2.1. Sample Preparation

Rice (*Oryza sativa* ssp.* japonica* cv. Nipponbare) seeds were germinated and grown by hydroponic culture in Yoshida's solution [1.425 mM NH_4_NO_3_, 0.323 mM NaH_2_PO_4_, 0.513 mM K_2_SO_4_, 0.998 mM CaCl_2_, 1.643 mM MgSO_4_, 0.009 mM MnCl_2_, 0.075 mM (NH_4_)_6_ Mo_7_O_24_, 0.019 mM H_3_BO_3_, 0.155 mM CuSO_4_, 0.036 mM FeCl_3_, 0.070 mM citric acid, and 0.152 mM ZnSO_4_] [5]. After 10 days, seedlings of uniform size and growth were subjected to Cd stress treatment by transferring them to a similar medium with 0.2, 1, or 50 *μ*M Cd. These values were chosen based on a report that the total dissolved Cd in 64 fields with Cd-contaminated soils ranged from 0.03 to 182 *μ*g/L [6] in previous experiences. The plants were maintained under Cd stress conditions for 14 d. Root and shoot samples were collected at approximately 9:00 AM, frozen in liquid nitrogen, and stored at −80°C until subsequent analyses. Total RNA was extracted from both root and shoot samples using an RNeasy Plant Kit (Qiagen, Hilden, Germany) according to the manufacturer's instructions. Construction of 34 cDNA libraries (2 tissues, 4 conditions, 2 treatments, and 2-3 replicates) from total RNA using a TruSeq RNA sample preparation kit and sequencing with the Illumina Genome Analyzer IIx (Illumina Inc., San Diego, CA, USA) was performed according to the manufacturer's protocols as a part of establishing TENOR (Transcriptome Encyclopedia of Rice, http://tenor.dna.affrc.go.jp/) [7]. The resulting RNA-Seq data were deposited in the DDBJ Sequence Read Archive (Accession number DRA000959).

### 2.2. Identification of Differentially Expressed Transcripts

The biological replicates (2-3) for each set of conditions were highly correlated (coefficient > 0.95), so reads from the same treatment were merged for subsequent analysis. Trimming of Illumina adaptor sequences and low-quality bases (*Q* < 20) by Cutadapt [8] and mapping of preprocessed reads to the IRGSP-1.0 genome assembly (http://rapdb.dna.affrc.go.jp/) were performed as described previously [9]. To estimate the expression levels of each transcript, all preprocessed reads were mapped to the IRGSP-1.0 genome assembly by Bowtie with default parameters [10]. The expression level for each transcript was calculated as the RPKM- (Reads per Kilobase Exon Model per million mapped reads-) derived read count [11] based on the number of uniquely mapped reads that overlapped with exonic regions. A *G*-test was performed to detect differentially expressed transcripts in the control and Cd treatments based on the statistical null hypothesis that the proportions of mapped reads to the transcripts were the same between the two conditions. A false discovery rate (FDR < 0.01) was used in multiple-hypothesis testing to correct for multiple comparisons. When calculating fold changes, 1 was added to avoid division by 0.

### 2.3. Hierarchical Clustering and Gene Ontology Enrichment Analysis

The Cd-responsive transcripts in root and shoot were used for hierarchical clustering analysis. We used the heatmap.2 in the R package gplots (version 2.11.0) to perform clustering analyses of transcripts. The *Z* scores were used to compare significant changes in gene expression. A Gene Ontology (GO) term was assigned to each transcript based on the GO annotations for biological process, molecular function, and cellular component in RAP-DB. GO enrichment was evaluated by Fisher's exact test with a FDR threshold of 5% for responsive transcripts in the biological process category of each cluster. The results were plotted as −log⁡10 of FDR values in a heatmap.

### 2.4. qRT-PCR Analysis

The expression of Cd upregulated genes in root sample was confirmed by qRT-PCR analysis. Rice seeds were germinated and grown in water in a growth chamber. After 10 days, the seedlings were subjected to different stress treatments by transferring them to water containing different reagents. RNA was extracted from them and the cDNA was synthesized according to the manufacturer's protocol and it is used for the further analysis as described previously [4]. The resulting cDNA was used for PCR amplification in the LightCycler 480 system (Roche, Basel, Switzerland) with each primer set (*Os04g0600300*: 5′-GGCGCTCTGAGAATCATCAC-3′, 5′-CATTCGGGAGCTCATCTCG-3′,* Os01g0692100*: 5′-ATTCACGAGTCCGCGATG-3′, 5′-CTCTCACCCGGATCACCC-3′,* Os12g0570700*: 5′-GCACTCATCTCAAGCTTTTC-3′, 5′-GCAAGACATCTTCTTGG-3′,* Os12g0571000*: 5′-ATTTCCTGAAGAGTTAAA-3′, 5′-TTCCGCAGCCGCAGCTTA-3′). The detection threshold cycle for each reaction was normalized using Ubiquitin1 primers (5′-CCAGGACAAGATGATCTGCC-3′, 5′-AAGAAGCTGAAGCATCCAGC-3′).

## 3. Results and Discussion

### 3.1. Low Cd Concentration Exposure of Rice Plants and Growth Retardation during the Treatment

**Figure 1:**
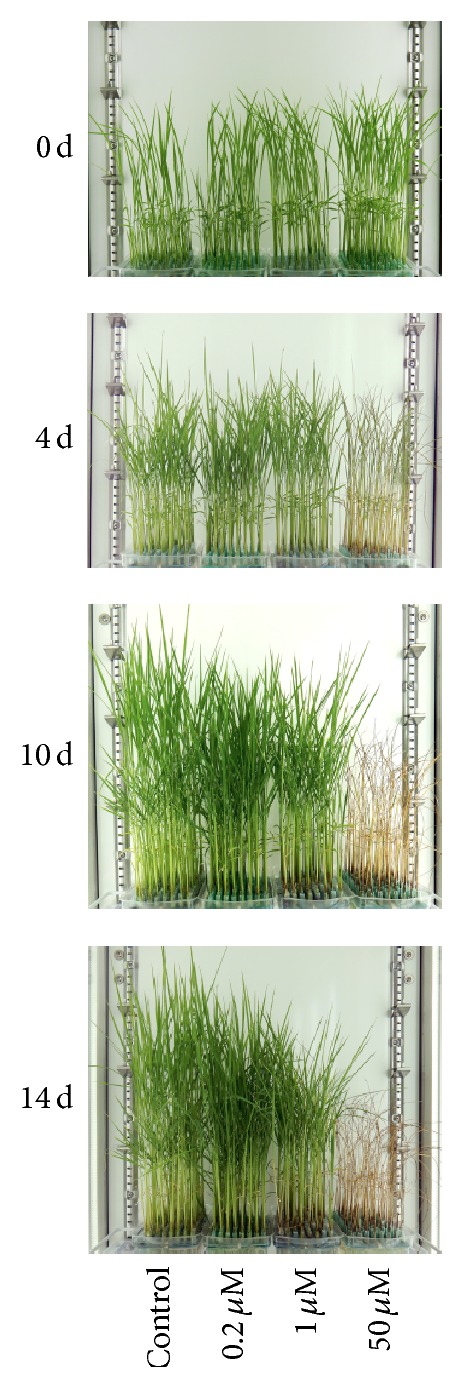
Phenotypic changes in rice plants grown in culture medium with low concentrations of Cd (0.2, 1 *μ*M) and a high concentration of Cd (50 *μ*M) from 0 to 14 d.

We used rice plants grown in hydroponic culture, which enabled us to control the Cd exposure easily. High Cd concentration exposure has been previously shown to elicit robust physiological responses and gene expression as acute toxic responses in rice seedlings [12–14]. Growth retardation of the shoot was slightly visible after 1 d (data not shown), the leaves turned yellow and the leaf tips of the seedlings began to wilt after 4 d, and all leaf blades were curled completely and the seedlings were wilting after 10 d under high Cd concentration (50 *μ*M) exposure (Figure 1). While no visible symptoms were observed in shoots under low Cd concentration exposure (0.2 and 1 *μ*M Cd) after 1 d, growth retardation occurred gradually compared with the control, with symptoms starting to appear after 7 d. Plants in the same growth chamber exposed to different Cd concentrations showed clear growth differences after 10 d (Figure 1). Even after 28 d, the seedlings under low Cd concentration exposure did not show yellow leaves or wilting (data not shown). These results suggested that high Cd concentration exposure causes fatal damage to plants while low Cd concentrations lead to growth retardation (Figure 1), which is supported by the fact that plant detoxification processes are insufficient to cope with this toxic metal beyond a 10 *μ*M dose [15].

### 3.2. Gene Expression Profiles under Low Cd Concentration Exposure in Rice

**Figure 2:**
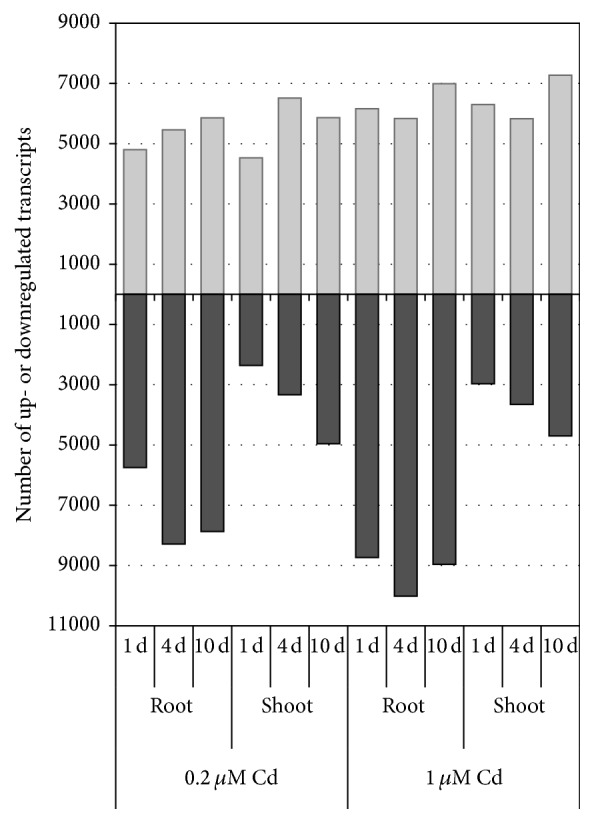
Distribution of upregulated and downregulated transcripts in roots and shoots in response to Cd exposure. RPKM fold changes at 1, 4, and 10 d were calculated for Cd-treated samples compared with nontreated samples (0 d). The total numbers of upregulated (upper) and downregulated (lower) transcripts in roots and shoots identified by RNA-Seq were determined by *G*-tests (FDR < 0.01) at each stress time point (1, 4, and 10 d) under 0.2 *μ*M (left) and 1 *μ*M (right) Cd exposure. The *x*-axis shows the time course and the *y*-axis shows the number of transcripts.

We next analyzed the transcriptome profiles of the response to Cd exposure using RNA-Seq during plant growth, at 1, 4, and 10 d after Cd treatment, and before treatment (0 d). For each set of conditions, an average of approximately 15.1 million (92.2%) quality-evaluated reads (total 211 million) were mapped to the rice genome sequence and used for further analysis (Table S1 in Supplementary Material available online at http://dx.doi.org/10.1155/2016/9739505). The number of upregulated transcripts ranged from 4,529 to 6,515, whereas the number of downregulated transcripts ranged from 2,359 to 8,734 under 0.2 *μ*M Cd (Figure 2). Twelve transcripts including GST, MT, and DREB (drought responsive element binding protein) 1E were upregulated more than 20-fold among the upregulated transcripts in roots at 0.2 *μ*M Cd. The number of upregulated transcripts ranged from 5,830 to 7,271 whereas the number of downregulated transcripts ranged from 2,965 to 10,020 under 1 *μ*M Cd (Figure 2). Fifty-one transcripts including GST, MT, Prx (peroxidase), and heat shock proteins were upregulated more than 20-fold among the upregulated transcripts in roots at 1 *μ*M Cd (Table 1). Induction of detoxification enzymes against oxidation stress such as GST and Prx under Cd exposure might be associated with the defense system that confers Cd tolerance to plants [16–18] even at low Cd concentrations. The cysteine-rich MT might function as a ligand for chelation of metal ions to defend against Cd toxicity [19]. The DREB/C-repeat binding factor (CBF) specifically interacts with the DRE/CRT cis-acting element and controls the expression of many stress-inducible genes in plants [20]. The activation of gene expression in several drought stress signal pathways under Cd exposure has been reported [4]. Five* heat shock proteins* (*Hsp*s) were strongly upregulated in roots under 1 *μ*M Cd, with the greatest relative expression at 1 d (Table 1). These genes may contribute to cellular homeostasis by protecting macromolecules such as enzymes, protein complexes, and membranes under Cd exposure. This result suggested that the roots of hydroponically cultured rice might be affected more directly and earlier by Cd exposure. There was a difference between the low Cd concentrations in that no* Hsps* were strongly upregulated in roots at 0.2 *μ*M Cd (Table 1), suggesting that the effect of this condition might be small or show time lag. In shoots, 15 and 11 transcripts were upregulated more than 20-fold among the upregulated transcripts under 0.2 and 1 *μ*M Cd, respectively (Table S2). Nine transcripts including Nramp1 (natural resistance-associated macrophage protein) were upregulated under both 0.2 and 1 *μ*M Cd (Table S2). In* Arabidopsis*, Nramp1 localizes to the plasma membrane and functions as a high-affinity transporter for manganese (Mn) uptake [21], while OsNramp5 uptakes Mn and Cd [22]. Transporters with heavy metal binding domains are often capable of transporting several metals, such as Fe, Zn, Mn, and Cd, because of their low substrate specificity [23–26]. We found that upregulation of a HLH DNA-binding domain containing transcription factor (*Os04g0301500*) in both roots and shoots peaked at 4 d under 0.2 *μ*M Cd; this protein may function as a regulatory factor under Cd exposure (Table 1, Table S2). The number of downregulated transcripts in roots peaked at 4 d after Cd exposure, while the number in shoots gradually increased under low Cd concentration exposure (Figure 2). A few dozen transcripts were downregulated less than 0.05-fold among the downregulated transcripts in roots and shoots under Cd exposure (Table S2). Therefore, a small part of transcripts were strongly up- or downregulated among several thousand responsive transcripts under low Cd concentration exposure. Large-scale changes in gene expression occurred in rice under Cd exposure, even at low concentrations, possibly because Cd is a nonessential metal for the plant.

To obtain a functional annotation of responsive transcripts under Cd exposure, we used GO biological process categories. The responsive transcripts in shoot and root were clustered into several groups based on their expression patterns. GO enrichment analysis was performed using clustered transcripts assigned by GO terms in RAP-DB (The Rice Annotation Project Database [http://rapdb.dna.affrc.go.jp/]) (Supplementary Figure S1). Enriched GO terms significantly in each cluster may represent the functional categories in rice under Cd exposure. Enriched GO terms of gradually upregulated transcripts under Cd exposure include metal ion transport (GO:0030001) (cluster 3 in root under 0.2 *μ*M Cd, cluster 4 in root under 1 *μ*M Cd), which may function in Cd transport. Response to oxidative stress (GO:0006979) and responsive to oxidative stress (GO:0006979) were also included in cluster 3 and cluster 4, respectively. This suggested that they might function in defense against Cd. Enriched GO terms of gradually downregulated transcripts under Cd exposure include translation (GO:0006412), translation elongation (GO:0006414), DNA replication (GO:0006260), and DNA repair (GO:0006281) (cluster 1 in root under 0.2 *μ*M Cd, cluster 2 in root under 1 *μ*M Cd). Photosynthesis, light harvesting (GO:0009765), and photosynthesis (GO:0015979) were also included in both clusters. These may function in plant growth. Thus, these correspond to the observed changes in phenotype (Figure 1), which clearly validated the RNA-Seq expression profiling data obtained from rice tissue under Cd stress condition. However, the pattern of gene expression is quite complex and would require more detailed analysis.

### 3.3. Constitutively Expressed Genes Responded Differently under Low Cd Concentration to High Cd Concentration

**Figure 3:**
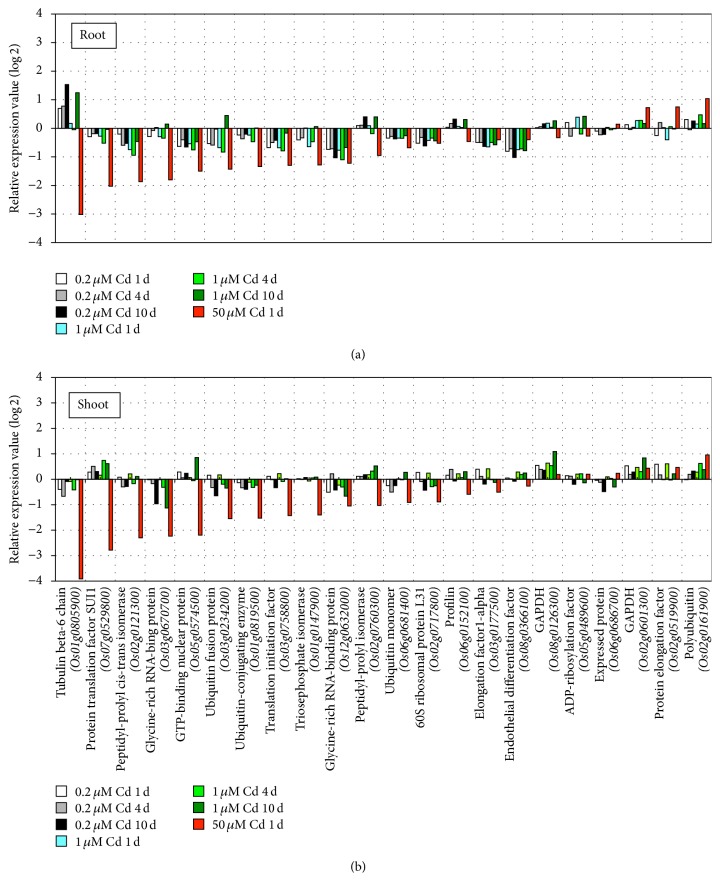
Response of constitutively expressed genes in roots and shoots to Cd exposure. The relative expression of constitutively expressed genes [27] in roots (a) and shoots (b) is shown under Cd exposure at each stress time point (1, 4, and 10 d) during 0.2 *μ*M (white, grey, and black) and 1 *μ*M (light blue, light green, and green) Cd exposure compared with nontreatment (0 d). The red bar shows the relative expression at 1 d under 50 *μ*M Cd exposure. The *x*-axis shows the genes and the *y*-axis shows relative expression. Wang et al. [27] suggested the following genes as candidates for constitutive expression: glycine-rich RNA-binding protein (*Os12g0632000*), expressed protein (*Os06g0686700*), profilin (*Os06g0152100*), ADP-ribosylation factor (*Os05g0489600*), triosephosphate isomerase (*Os01g0147900*), glycine-rich RNA-binding protein (*Os03g0670700*), peptidyl-prolyl cis-trans isomerase (*Os02g0121300*), endothelial differentiation factor (*Os08g0366100*), ubiquitin monomer (*Os06g0681400*), protein translation factor SUI1 (*Os07g0529800*), GAPDH (*Os08g0126300*), polyubiquitin (*Os02g0161900*), protein elongation factor (*Os02g0519900*), translation initiation factor (*Os03g0758800*), ubiquitin-conjugating enzyme (*Os01g0819500*), GTP-binding nuclear protein (*Os05g0574500*), peptidyl-prolyl isomerase (*Os02g0760300*), and 60S ribosomal protein L31 (*Os02g0717800*). Their paper also introduced the following genes that have frequently been used as internal controls in expression analyses: elongation factor1-alpha (*Os03g0177500*), ubiquitin fusion protein (*Os03g0234200*), GAPDH (*Os02g0601300*), and tubulin beta-6 chain (*Os01g0805900*).

As many genes responded to both low and high Cd concentrations [4], we assessed the effect of the stress degree on rice seedlings through the expression of constitutively expressed genes. We investigated the expression of 18 genes annotated by the RAP that were expressed constitutively in 39 tissues collected throughout the life cycle of the rice plant from two varieties according to 190 Affymetrix GeneChip Rice Genome Arrays, in addition to four genes annotated by the RAP that have frequently been used as internal controls in expression analyses [27]. The results showed that the expression of more than half of them fluctuated drastically (>2 or <2) in roots or shoots after 1 d of high Cd concentration exposure (Figure 3). This drastic response may be partly because RNA-Seq can accurately quantify gene expression levels over a broad dynamic range with high resolution and sensitivity [10, 28, 29]. However, our results suggest that their expression is greatly affected by strong stress, even though they are expressed constitutively across the developmental course. Note that a high Cd concentration can cause fatal damage to rice seedlings, such as by affecting homeostasis, which corresponds to the observed changes in phenotype (Figures 1 and 3).

### 3.4. Comparative Gene Expression Analysis between Low and High Cd Concentrations Reveals Novel Cd-Responsive Transporters

**Figure 4:**
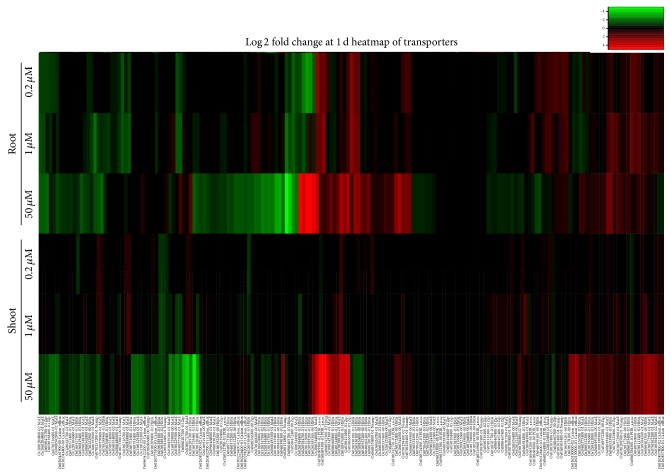
Expression profiling of metal ion transporter genes in roots and shoots under Cd exposure at 1 d demonstrates Cd concentration-dependent differences. Heatmap analysis of metal ion transporters containing Pfam domains [PF01554 (MatE), PF08370 (PDR_assoc), PF01545 (Cation_efflux), PF02535 (Zip), PF00403 (HMA), and PF01566 (Nramp)]. The relative expression values under 0.2, 1, and 50 *μ*M Cd (data from [4]) are presented. The color scale shows log2-transformed transcript levels for each gene.

We investigated the expression of metal transporter genes containing metal ion binding Pfam domains [PF01554 (MatE), PF08370 (PDR_assoc), PF01545 (Cation_efflux), PF02535 (Zip), PF00403 (HMA), and PF01566 (Nramp)] that may function in Cd transport under Cd exposure. The expression of 183 transport transcripts was compared between low and high Cd concentration treatments in roots and shoots at 1 d, because Cd uptake from the hydroponic culture and efflux pumping are initial responses to Cd exposure (Figure 4, Table S3). The transcripts tended to be more responsive in roots and shoots under higher Cd concentration exposure. This result indicated the potential of the RNA-Seq strategy to reveal novel Cd-responsive transporters by analyzing gene expression under exposure to different Cd concentrations. The responsive transcripts might function in roots at the early stage of Cd exposure. No transcripts were upregulated more than 3-fold in shoots under low Cd exposure (Figure 4, Table S3), suggesting that the effect takes more time to appear in shoots.* Os03g0667500* (Zip, root) encoding iron-regulated transporter 1 (IRT1) was upregulated more than 5-fold under low Cd concentrations but responded only slightly under the high Cd concentration. IRT1s often transport Cd because of their low substrate specificity [24–26, 30].* Os02g0585200* (HMA, root),* Os03g0152000* (HMA, root),* Os0g0584800* (HMA, root),* Os01g0609900* (PDR_assoc, shoot), and* Os01g0609300* (PDR_assoc, shoot) showed the highest (32-fold) upregulation under high Cd concentration exposure and responded only slightly to low Cd concentrations (Table S3). The balance between Cd and various other metal ions in the hydroponic culture might affect the expression of these genes, because specific systems for transporting Cd may have not developed in rice as it is a nonessential metal. The effects of other ions on the expression of transporters [4] and responsive genes associated with defense systems against Cd (Supplementary Figure S2) have been indicated.

## 4. Conclusions

We generated gene expression profiles for rice seedlings grown under low Cd concentrations. Phenotypic observations and constitutive gene expression indicated that low Cd concentrations cause growth retardation but are far from being fatal in rice. Several genes associated with defense systems were strongly upregulated; the expression of metal ion transporter genes tended to correlate with Cd concentration and GO enrichment analysis of the clustered genes based on their expression patterns, suggesting that our transcriptome profiles reflect responses to Cd in rice. Our data also suggest that it could be dangerous to eat plants that do not show specific Cd pollution symptoms growing in soil contaminated by small amounts of Cd. Establishing the exact composition and organization of the transcriptional network underlying the response to Cd exposure will provide a robust tool for improving crops in the future, for example, by creating low Cd uptake plants.

## Supplementary Material

Mapping of RNA-seq reads obtained from root and shoot samples to the reference IRGSP-1.0 genome sequence.

## Figures and Tables

**Table 1:** Cadmium-upregulated transcripts identified in roots by RNA-Seq analysis.

Transcript	Description	Fold change					
		Root			Shoot		
		1 d	4 d	10 d	1 d	4 d	10 d
0.2 *µ*M Cd							
***Os10t0527400-01***	Tau class GST protein 3	27.8	21.4	27.5	1.2	2.0	1.7
*Os03t0283000-00*	In2-1 protein	27.5	2.8	1.0	1.3	1.1	1.5
***Os08t0156000-01***	Conserved hypothetical protein	26.4	21.4	25.3	1.3	1.6	1.7
***Os01t0627967-00***	Hypothetical protein	26.1	16.5	24.1	1.5	1.9	1.4
***Os04t0178300-02***	Syn-copalyl diphosphate synthase	20.1	8.0	20.3	0.6	4.2	1.4
*Os04t0301500-01*	HLH (helix-loop-helix) DNA-binding domain containing protein	0.4	33.1	0.5	1.0	47.5	9.2
*Os02t0676800-01*	DREB1E (drought responsive element binding protein 1E)	0.9	28.7	0.9	1.2	10.9	2.0
*Os02t0179200-01*	Glutamine amidotransferase class-I domain containing protein	0.8	28.1	1.7	0.9	3.2	1.1
*Os12t0154800-00*	RmlC-like jelly roll fold domain containing protein	4.0	21.4	5.7	1.0	1.4	1.2
***Os12t0570700-01***	MT (metallothionein)-like protein type 1	18.6	20.3	15.8	0.9	1.0	0.9
***Os03t0836800-01***	IAA-amino acid hydrolase 1	4.3	6.5	33.6	1.0	1.0	1.0
*Os10t0333700-00*	Plant disease resistance response protein domain containing protein	9.7	6.0	21.6	1.0	1.0	1.0
1 *µ*M Cd							
***Os04t0178300-02***	Syn-copalyl diphosphate synthase	122.0	32.1	25.5	0.5	1.0	3.6
*Os04t0178300-01*	Isoform 3 of Syn-copalyl diphosphate synthase	109.8	27.8	21.5	0.5	0.9	3.1
*Os04t0178400-01*	Cytochrome P450 CYP99A1	69.8	21.1	16.0	0.8	1.0	2.8
*Os03t0267000-00*	Heat shock protein 180	57.5	7.7	10.9	1.2	0.7	0.7
*Os03t0266900-01*	Heat shock protein 173	47.0	4.9	5.3	1.0	0.4	0.6
*Os01t0136200-01*	Heat shock protein 1	43.7	3.9	1.3	1.0	1.0	1.0
*Os07t0190000-01*	1-Deoxy-D-xylulose 5-phosphate synthase 2 precursor	42.4	11.5	8.6	0.7	1.1	3.9
*Os07t0127500-01*	PR-1a pathogenesis related protein precursor	40.0	5.6	5.0	0.8	0.8	2.1
*Os07t0154100-01*	Viviparous-14	38.8	5.2	1.5	1.1	1.4	2.3
*Os07t0154201-00*	Hypothetical gene	37.7	4.7	1.3	1.0	1.3	2.1
*Os12t0555200-01*	Probenazole-inducible protein PBZ1	37.7	13.5	10.9	0.3	0.5	2.2
*Os06t0586000-01*	Conserved hypothetical protein	37.6	9.3	6.5	0.6	0.9	1.4
***Os10t0527400-01***	Tau class GST protein 3	34.3	18.0	32.4	1.1	1.4	2.0
*Os12t0555000-01*	Probenazole-inducible protein PBZ1	33.2	13.5	11.0	0.6	0.7	2.5
*Os03t0277700-01*	Protein of unknown function DUF26 domain containing protein	32.8	7.6	3.4	1.0	0.6	1.0
*Os11t0687100-01*	von Willebrand factor (type A domain)	32.5	4.1	13.8	0.7	0.7	2.3
*Os05t0211700-00*	—	28.8	1.4	1.2	1.0	1.0	1.0
*Os06t0662550-01*	Conserved hypothetical protein	28.5	7.8	8.8	0.8	0.8	1.6
*Os01t0944100-02*	Conserved hypothetical protein	28.4	6.3	9.8	0.5	0.6	1.7
*Os06t0568600-01*	Ent-kaurene oxidase 1	27.1	28.1	11.0	0.6	1.4	4.7
*Os12t0418600-01*	Hypothetical conserved gene	26.7	2.0	1.3	1.0	1.0	1.0
*Os12t0258700-01*	Cupredoxin domain containing protein	26.2	14.7	10.6	0.7	1.1	7.1
*Os01t0615100-01*	Substilin/chymotrypsin-like inhibitor	25.6	9.5	7.9	0.7	1.0	1.8
*Os04t0107900-02*	Heat shock protein 81-1	25.6	2.5	1.6	1.0	1.0	0.9
*Os09t0493000-01*	Conserved hypothetical protein	25.3	2.6	1.8	0.9	1.2	0.9
***Os01t0627967-00***	Hypothetical protein	25.3	19.5	21.6	1.3	1.8	1.4
*Os01t0944100-03*	Conserved hypothetical protein	25.2	4.6	6.3	0.6	0.6	1.8
*Os04t0180400-01*	Cytochrome P450 99A2	24.4	4.3	6.0	0.5	0.5	3.1
*Os04t0108101-00*	Hypothetical protein	24.4	2.3	1.4	1.0	1.0	1.0
*Os02t0269600-00*	Subtilase	22.6	7.8	4.1	0.3	1.2	6.0
*Os01t0136000-00*	Heat shock protein 175	22.5	3.1	1.2	1.0	1.4	1.2
*Os04t0180500-00*	Hypothetical protein	22.2	4.0	5.4	0.5	0.6	3.1
*Os01t0946600-01*	Conserved hypothetical protein	21.8	16.6	8.0	0.7	0.7	0.8
*Os09t0255400-02*	Indole-3-glycerol phosphate synthase	21.4	5.1	3.8	0.7	0.9	2.3
*Os01t0348900-01*	SalT gene product	21.2	6.5	8.9	0.1	0.1	0.2
*Os12t0491800-01*	Ent-kaurene synthase 1A	21.1	1.5	1.7	0.4	0.8	5.5
*Os01t0132000-01*	Wound-induced protease inhibitor	21.0	8.8	11.6	1.6	0.5	0.2
*Os11t0592200-01*	Chitin-binding allergen Bra r 2	20.7	3.4	2.8	0.7	0.5	1.6
*Os01t0963000-01*	Prx (Peroxidase) BP 1 precursor	20.6	3.8	4.4	0.7	1.1	1.3
*Os08t0189600-01*	Oryza sativa germin-like protein 8-7	20.6	11.5	6.7	2.1	1.5	0.8
*Os07t0496250-01*	Expansin-like B1	20.5	2.2	2.2	1.5	1.2	4.5
*Os01t0963000-04*	Prx (Peroxidase) BP 1 precursor	20.3	3.7	4.4	0.7	1.1	1.3
*Os09t0255400-01*	Indole-3-glycerol phosphate synthase	20.2	5.2	3.7	0.7	0.9	2.3
*Os11t0601950-01*	cDNA clone:002-114-B06	20.0	1.7	1.9	0.7	1.0	1.1
*Os03t0129400-01*	Hypothetical protein	10.3	27.1	17.6	1.0	1.9	3.6
*Os01t0322700-01*	Nonprotein coding transcript	12.2	25.5	15.7	0.9	1.3	2.5
*Os03t0129400-02*	EST AU078206 corresponds to a region of the predicted gene	9.4	24.3	16.3	1.1	1.4	2.8
***Os12t0570700-01***	MT (metallothionein)-like protein type 1	16.7	21.2	17.7	0.8	0.8	3.1
*Os12t0571000-01*	MT (metallothionein)-like protein type 1	13.9	20.0	13.0	0.9	1.0	3.6
***Os08t0156000-01***	Conserved hypothetical protein	15.4	17.9	26.0	1.1	1.5	1.6
***Os03t0836800-01***	IAA-amino acid hydrolase 1	0.7	4.0	23.7	1.0	1.0	1.0

Reads were mapped to the rice genome and responsive genes were identified by *G*-tests. Transcripts upregulated more than 20-fold in one or more treatments/time points in roots are shown. Transcripts in bold were upregulated under both 1 and 0.2 *μ*M Cd exposure.
