# Supplementary material for: Genome-Wide Transcriptome Analysis of Cadmium Stress in Rice
Source: Biomed Res Int. 2016 Feb 29;2016:9739505. doi: 10.1155/2016/9739505 (PMC4789393; doi:10.1155/2016/9739505)
Supplement: Supplementary file 1 — Mapping of RNA-seq reads obtained from root and shoot samples to the reference IRGSP-1.0 genome sequence. [file 9739505.f1.pdf]

A

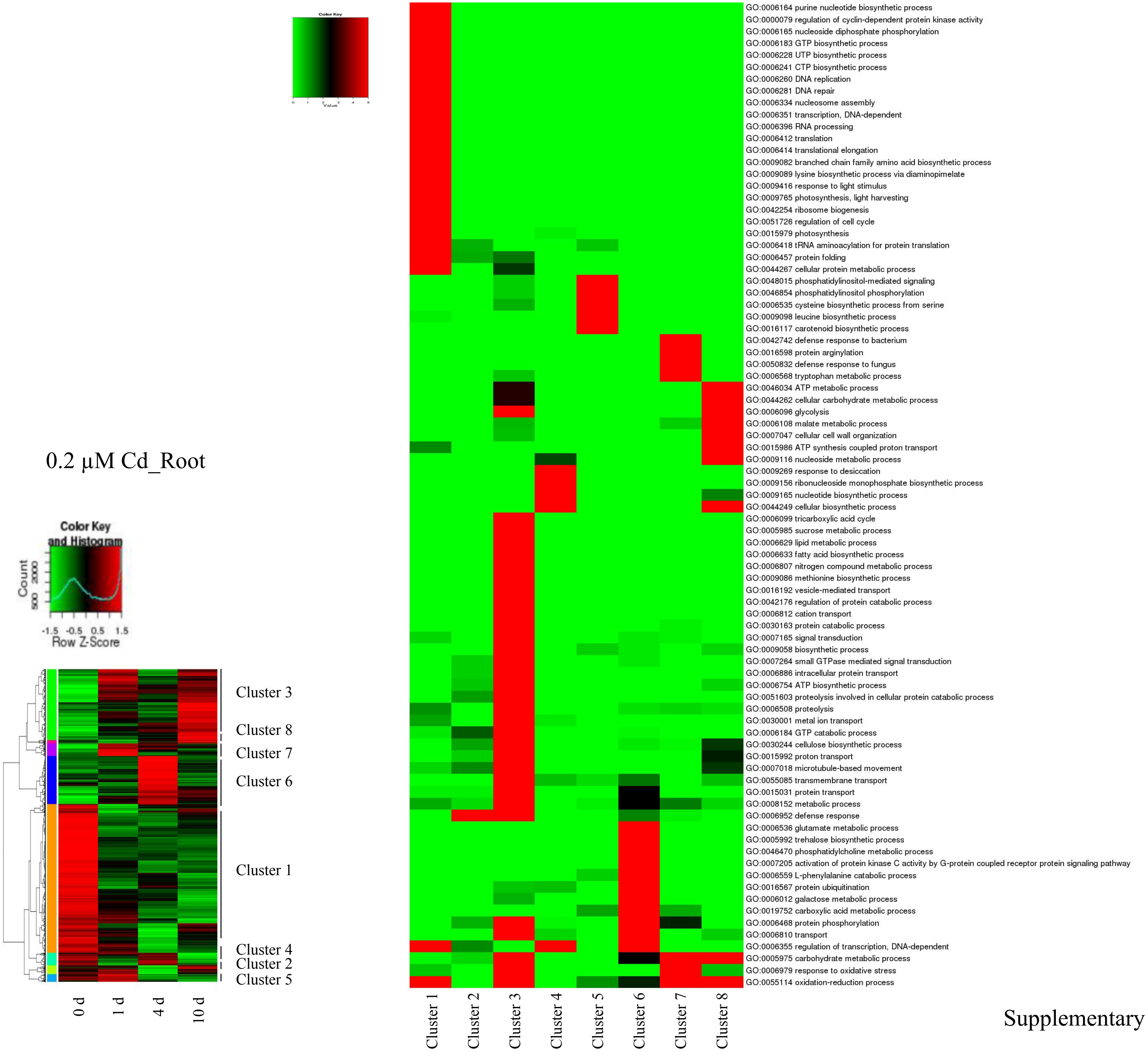

Supplementary Figure S1

B

0.2 µM Cd_Shoot

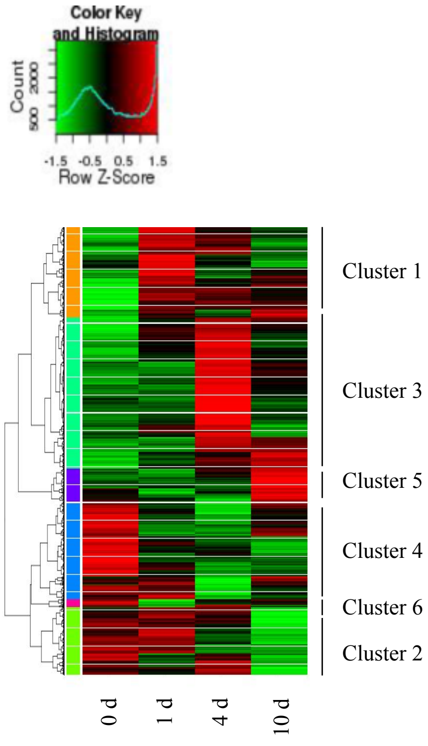

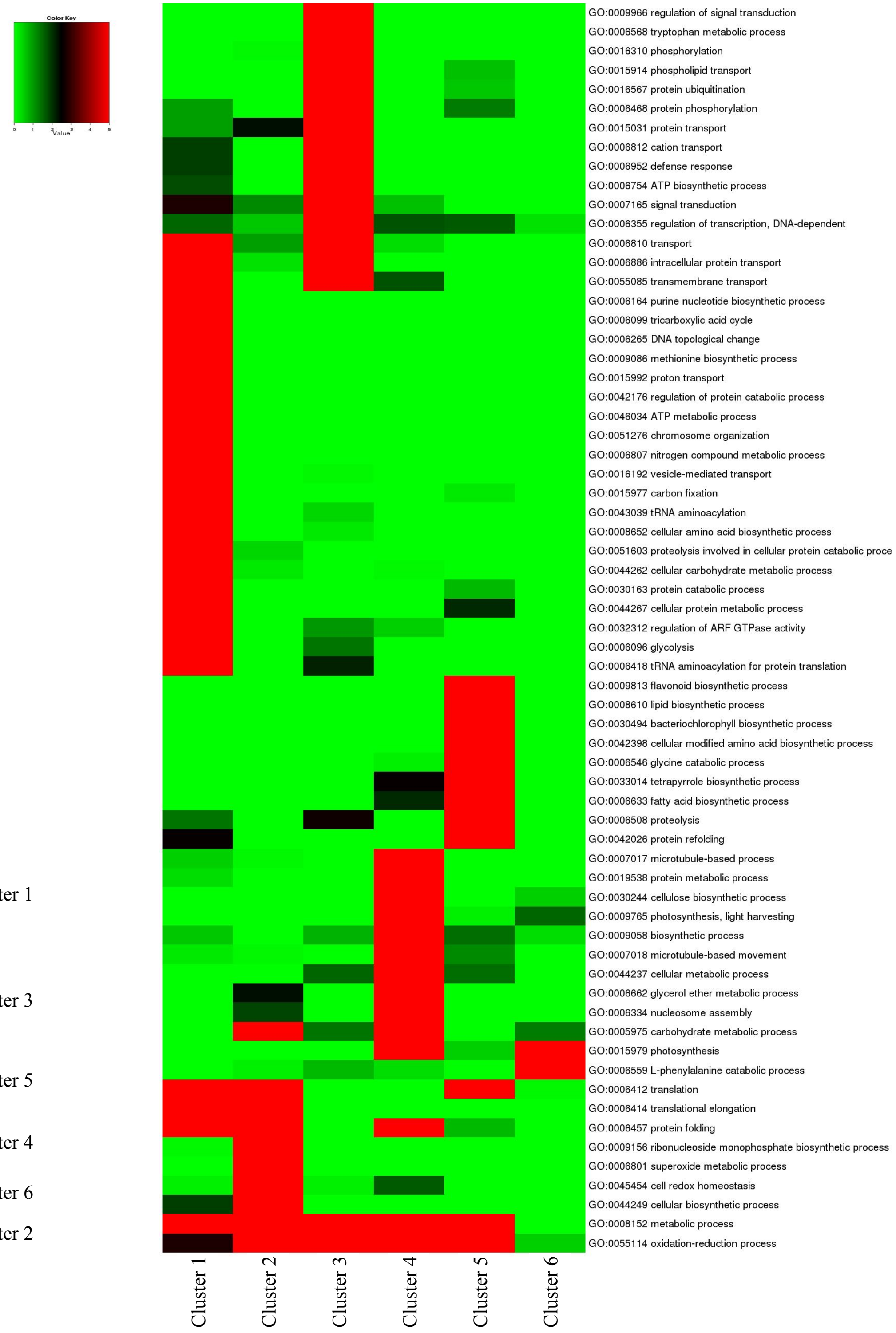

Supplementary Figure S1

C

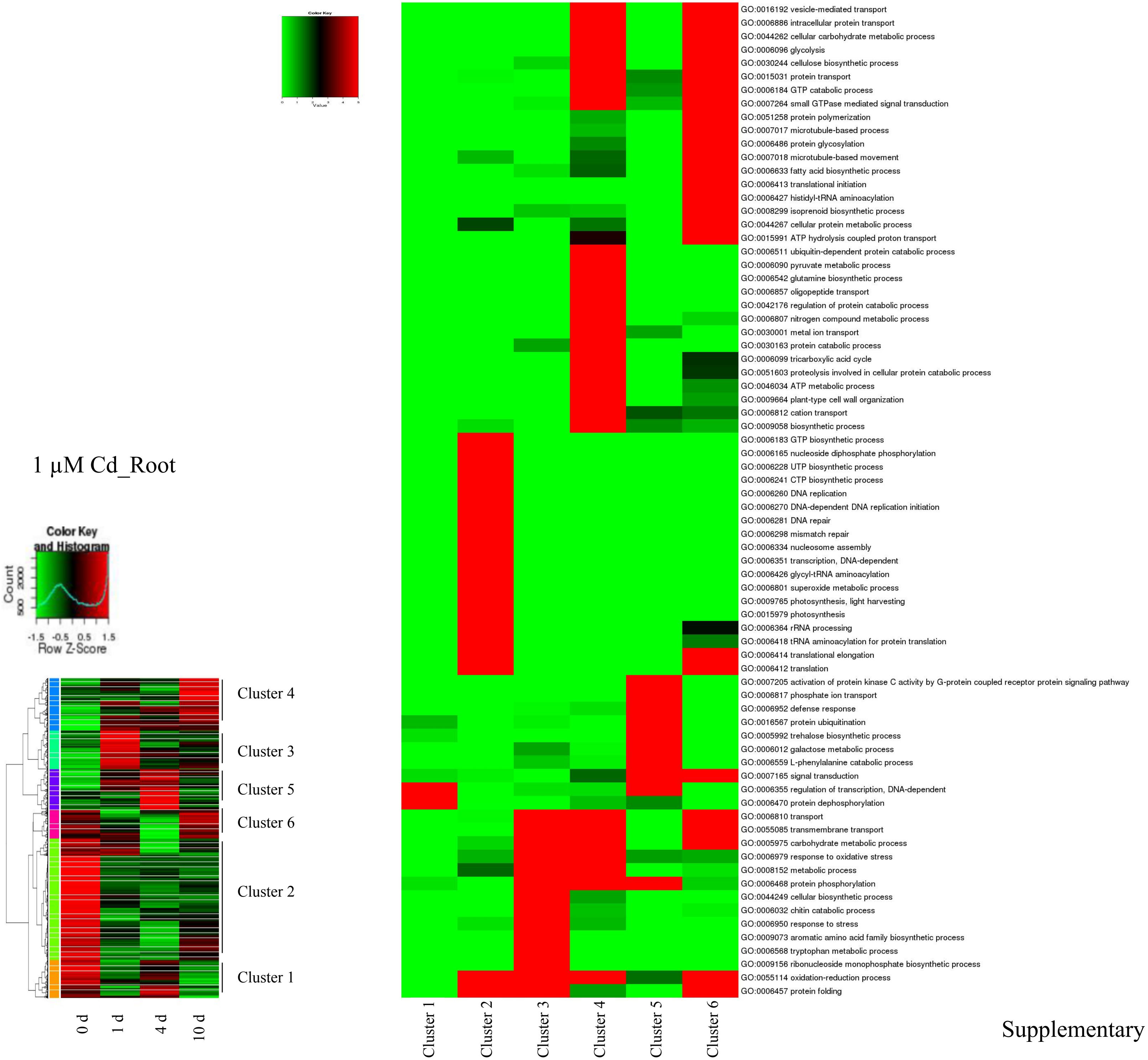

Supplementary Figure S1

D

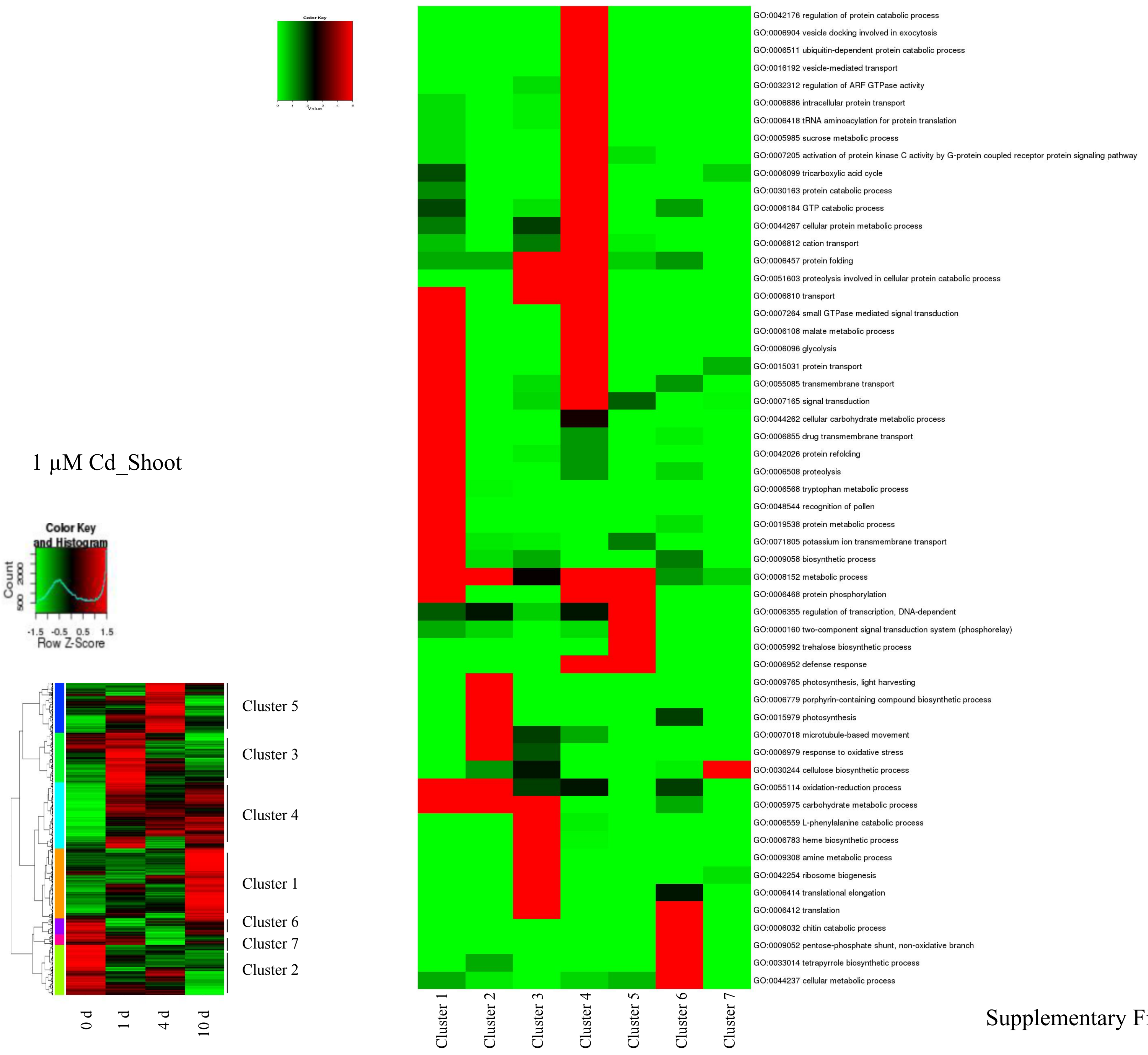

Supplementary Figure S1

A

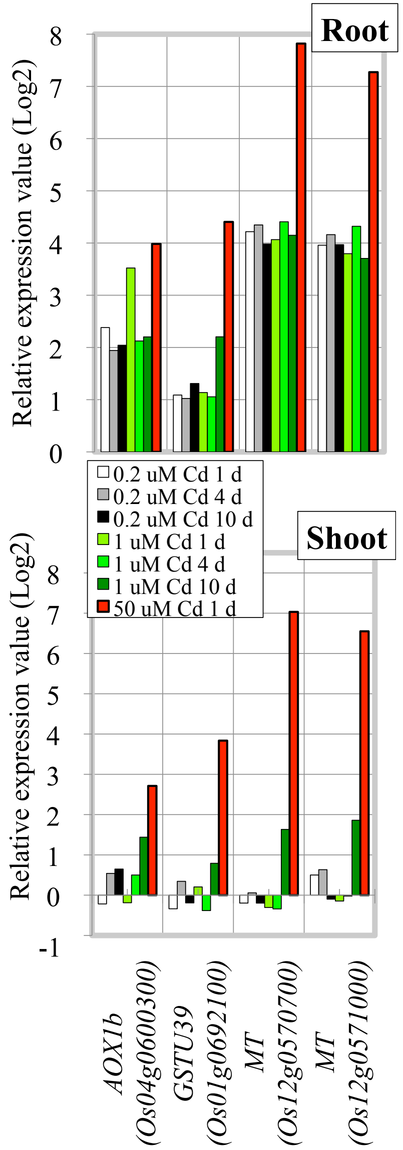

B

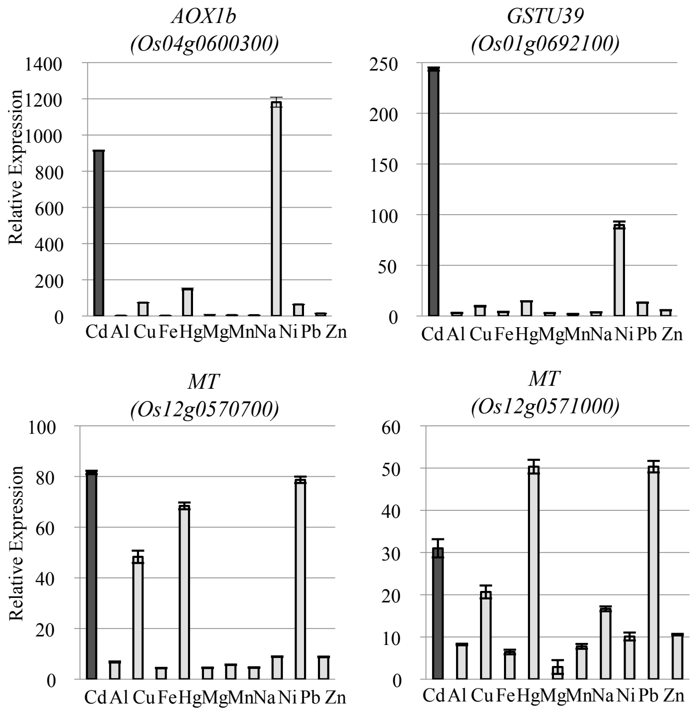

Supplementary Figure S2

Supplementary Table S1. Mapping of RNA-Seq reads obtained from root and shoot samples to the reference IRGSP-1.0 genome sequence.

Sample			Preprocessed reads	Aligned	%	Unique non junc	%	Unique junc	%	Multi	%	Unaligned	%
Root	Control	0 d	27583462	23866366	86.52	17979889	65.18	4914667	17.82	971810	3.52	3717096	13.48
	0.2 µM Cd	1 d	9988187	8303026	83.13	6120296	61.28	1857792	18.60	324938	3.25	1685161	16.87
		4 d	10796972	9651791	89.39	7126843	66.01	2166457	20.07	358491	3.32	1145181	10.61
		10 d	10946913	9261544	84.60	6717523	61.36	2190187	20.01	353834	3.23	1685369	15.40
	1 µM Cd	1 d	11963408	10655539	89.07	7904136	66.07	2339255	19.55	412148	3.45	1307869	10.93
		4 d	11104337	10253176	92.33	7673423	69.10	2214831	19.95	364922	3.29	851161	7.67
		10 d	23128192	18958499	81.97	13790354	59.63	4461941	19.29	706204	3.05	4169693	18.03
Shoot	Control	0 d	28320011	27672810	97.71	20666053	72.97	6063038	21.41	943719	3.33	647201	2.29
	0.2 µM Cd	1 d	11361926	11078040	97.50	7990472	70.33	2627344	23.12	460224	4.05	283886	2.50
		4 d	10075073	9835486	97.62	7150808	70.98	2341532	23.24	343146	3.41	239587	2.38
		10 d	28595535	28014745	97.97	20391652	71.31	6619292	23.15	1003801	3.51	580790	2.03
	1 µM Cd	1 d	12386946	12097953	97.67	8726675	70.45	2881805	23.26	489473	3.95	288993	2.33
		4 d	14603307	14296796	97.90	10363189	70.96	3424710	23.45	508897	3.48	306511	2.10
		10 d	17498911	17093191	97.68	12285165	70.21	4253212	24.31	554814	3.17	405720	2.32

Supplementary Table S2. Cadmium up- and downregulated transcripts identified in roots and shoots by RNA-Seq analysis.

Transcript	Description	Fold change					
		Root			Shoot		
		1 d	4 d	10 d	1 d	4 d	10 d
Upregulated transcripts in shoots							
0.2 μM Cd							
Os04t0301500-01	HLH (helix-loop-helix) DNA-binding domain containing protein	0.4	33.1	0.5	1.0	47.5	9.2
Os01t0162200-00	Leucine-rich repeat domain containing protein	1.1	2.4	1.0	1.4	24.5	2.1
Os01t0162400-01	Conserved hypothetical protein	1.0	1.1	1.0	1.3	24.1	2.1
Os11t0151400-01	Cytochrome P450 family protein	0.9	15.5	1.1	0.9	22.2	5.5
Os01t0162300-01	Leucine-rich repeat, plant specific containing protein	1.0	3.0	0.9	1.0	22.2	1.8
Os03t0183500-01	Protein of unknown function DUF581 family protein	1.0	13.0	0.7	2.2	21.0	6.5
Os07t0142100-00	Conserved hypothetical protein	0.9	1.1	0.9	1.0	1.0	213.6
Os01t0647200-04	Non-protein coding transcript	0.8	1.7	1.3	0.4	0.5	83.9
Os01t0647200-01	Hypothetical conserved gene	0.9	2.0	1.3	0.7	0.8	61.2
Os10t0195250-01	Conserved hypothetical protein	3.7	6.8	7.7	1.0	1.3	54.9
Os07t0258400-02	Nramp1	2.3	2.2	3.4	1.4	1.0	48.5
Os07t0258400-01	Nramp1	2.3	2.3	3.5	1.2	0.9	42.5
Os01t0952800-01	Achaete-scute transcription factor related domain containing protein	2.6	4.3	6.1	1.0	5.1	32.2
Os11t0262600-00	Conserved hypothetical protein	2.0	5.8	8.0	1.0	1.0	28.5
Os12t0236225-00	Conserved hypothetical protein	2.7	6.2	6.8	0.9	0.9	23.0
1 μM Cd							
Os07t0142100-00	Conserved hypothetical protein	0.9	1.1	0.9	1.2	1.4	302.6
Os01t0647200-04	Non-protein coding transcript	0.4	1.4	1.3	0.4	0.9	93.4
Os01t0647200-01	Hypothetical conserved gene	0.5	1.3	1.4	0.7	0.9	72.3
Os10t0195250-01	Conserved hypothetical protein	2.9	5.0	6.4	0.9	1.0	62.4
Os07t0258400-02	Nramp1	1.5	2.8	2.5	1.1	1.1	56.2
Os07t0258400-01	Nramp1	1.6	2.8	2.7	1.0	1.0	49.7
Os05t0202800-00	MT (metallothionein)-like protein 3B	1.0	5.3	2.4	1.0	4.7	39.6
Os01t0952800-01	Achaete-scute transcription factor related domain containing protein	1.7	5.1	3.9	1.0	1.7	29.3
Os11t0262600-00	Conserved hypothetical protein	1.9	5.4	5.7	1.0	1.0	27.3
Os12t0236225-00	Conserved hypothetical protein	1.8	3.9	4.2	0.9	0.9	21.8
Os01t0155800-01	Conserved hypothetical protein	2.8	6.4	8.3	0.4	0.5	21.0
Downregulated transcripts in roots							
0.2uM Cd							
Os02t0585700-00	Quinonprotein alcohol dehydrogenase-like domain containing protein	0.05	0.00	0.00	1.35	1.00	1.00
Os02t0585650-01	Hypothetical gene	0.06	0.00	0.00	1.39	0.90	1.09
Os02t0586000-01	Quinonprotein alcohol dehydrogenase-like domain containing protein	0.06	0.00	0.00	1.34	1.10	0.95
Os03t0259500-00	Ubiquitin	0.67	0.03	0.03	1.28	1.00	1.16
Os09t0483200-00	Ubiquitin/ribosomal fusion protein	0.60	0.03	0.09	1.00	1.00	1.07
Os03t0259551-00	Non-protein coding transcript	0.69	0.04	0.04	1.50	1.00	1.10
Os10t0386300-01	Conserved hypothetical protein	1.40	0.05	0.07	1.09	1.00	1.00
Os01t0304400-00	Conserved hypothetical protein	0.07	0.05	0.12	0.63	0.51	0.80
Os09t0483150-00	Hypothetical gene	0.47	0.05	0.08	1.00	1.00	1.10
1uM Cd							
Os02t0585700-00	Quinonprotein alcohol dehydrogenase-like domain containing protein	0.00	0.00	0.00	1.00	1.00	1.00
Os02t0585650-01	Hypothetical gene	0.01	0.00	0.00	0.90	0.90	0.90
Os09t0452700-00	Ubiquitin	0.01	0.04	0.03	1.44	1.09	1.00
Os02t0586000-01	Quinonprotein alcohol dehydrogenase-like domain containing protein	0.01	0.00	0.00	1.08	0.95	0.95
Os03t0259551-00	Non-protein coding transcript	0.02	0.02	0.04	1.00	1.00	1.00
Os09t0483200-00	Ubiquitin/ribosomal fusion protein	0.02	0.05	0.06	1.00	1.00	1.00
Os09t0452750-00	Non-protein coding transcript	0.02	0.05	0.04	1.00	1.20	1.00
Os03t0259500-00	Ubiquitin	0.02	0.02	0.04	1.00	1.00	1.00
Os09t0483350-00	Non-protein coding transcript	0.03	0.07	0.10	0.77	0.79	0.47
Os11t0428800-01	Cupredoxin domain containing protein	0.03	0.32	0.14	1.00	1.38	1.40
Os12t0291100-02	Ribulose bisphosphate carboxylase small chain	0.04	0.09	0.07	0.45	0.48	0.57
Os09t0483400-01	Ubiquitin/ribosomal fusion protein	0.04	0.08	0.08	1.39	0.86	1.02
Os03t0307300-01	Nicotianamine synthase 1	0.04	0.64	1.22	1.00	1.00	1.17
Os09t0483150-00	Hypothetical gene	0.05	0.08	0.06	1.00	1.00	1.00
Os12t0291100-01	Petunia ribulose 1,5-bisphosphate carboxylase small subunit	0.05	0.09	0.08	0.54	0.54	0.64
Os07t0625500-01	Fimbriata-associated protein	0.05	0.04	0.08	1.00	1.00	1.00
Os03t0103200-01	Physical impedance induced protein	0.50	0.02	0.06	1.18	1.91	0.78
Os10t0386300-01	Conserved hypothetical protein	0.06	0.05	0.08	1.17	1.00	1.00
Os03t0103300-00	QLTG-3-1 protein	0.17	0.05	0.08	1.19	1.07	0.72
Os08t0536341-00	Non-protein coding transcript	0.21	0.05	0.04	0.56	0.63	0.67
Os04t0538400-01	Nodulin 21	0.05	0.08	0.05	1.22	0.70	1.13
Downregulated transcripts in shoots							
1uM Cd							
Os03t0658800-03	Cytochrome P450 family protein	0.62	0.69	0.62	0.12	0.05	0.02
Os03t0658800-01	Cytochrome P450 family protein	0.63	0.69	0.63	0.12	0.05	0.03
Os05t0190300-01	Acid phosphatase (Class B) family protein	1.00	1.00	1.05	0.34	0.14	0.02
Os10t0409400-01	Polygalacturonase isoenzyme 1 beta subunit precursor	0.90	0.97	0.90	0.91	0.09	0.03
Os06t0473000-01	Hypothetical conserved gene	0.47	0.47	0.76	0.27	0.22	0.04
Os11t0645400-01	Plant disease resistance response protein family protein	0.89	1.81	5.08	0.25	0.11	0.04

Reads were mapped to the rice genome and responsive genes were identified by G-tests. Transcripts upregulated more than 20-fold or downregulated less than 0.05-fold in one or more treatments/time points in root and shoot are shown. Transcripts in bold were upregulated under both 1 and 0.2 μM Cd exposure.

Supplementary Table S3. Gene expression of metal ion transporters identified in rice.

Family	Transcript	Fold change					
		Root			Shoot		
		0.2 µM	1 µM	50 µM	0.2 µM	1 µM	50 µM
Cation_efflux	*Os01t0130000-01*	0.7	0.7	1.0	1.0	1.0	**6.7**
	Os08t0422200-00	1.0	1.0	1.0	1.0	1.0	0.6
	Os02t0775100-01	0.8	0.6	1.0	1.0	1.2	0.5
	Os01t0130000-02	0.7	0.6	0.9	1.0	1.0	**5.8**
	Os05t0128400-01	1.5	1.4	0.9	1.2	1.5	1.0
	Os01t0837800-01	1.3	1.4	0.7	1.3	1.4	3.6
	Os01t0837800-02	1.3	1.4	0.7	1.3	1.4	3.4
	Os05t0461900-00	1.0	1.0	0.5	1.0	1.0	0.6
	Os03t0346800-00	1.0	1.0	0.5	1.0	1.0	0.7
	Os02t0832700-01	1.0	1.0	0.5	1.0	1.2	0.8
	Os02t0832700-02	1.0	1.0	0.4	1.0	1.0	0.7
	Os04t0298200-01	0.8	0.8	0.4	1.0	0.8	1.2
	Os03t0226400-01	1.0	1.0	0.3	1.0	0.8	1.2
	Os03t0226400-02	1.0	1.0	0.3	1.0	0.8	1.2
HMA	*Os02t0585200-01*	0.1	1.9	**32.0**	1.0	1.0	2.1
	Os03t0152000-01	0.1	0.6	**32.0**	1.0	1.0	1.0
	Os02t0584800-01	0.4	0.5	**32.0**	1.0	0.5	0.8
	Os02t0585100-00	0.3	0.6	**23.2**	1.0	1.0	**5.2**
	Os02t0584700-01	0.2	0.4	**17.5**	1.0	1.0	1.0
	Os03t0372600-00	1.0	1.0	**9.9**	1.0	1.0	2.4
	Os02t0530100-02	3.6	4.2	**7.2**	1.4	1.0	0.4
	Os02t0530100-01	3.4	3.9	**7.0**	1.0	1.0	0.4
	Os01t0976300-01	1.0	1.5	**6.4**	1.0	1.0	**13.8**
	Os04t0244800-01	1.0	1.0	3.7	1.0	1.6	**5.6**
	Os06t0542300-01	2.0	1.0	2.4	1.0	1.0	**15.2**
	Os06t0665800-01	0.8	0.7	2.3	1.0	1.4	**5.9**
	Os04t0533900-01	2.1	3.2	4.3	1.0	1.0	2.8
	Os04t0661100-00	1.0	1.0	4.2	1.0	1.0	1.0
	Os01t0826000-00	1.0	1.0	3.8	1.0	1.0	0.5
	Os10t0209700-01	1.0	1.0	3.3	1.3	1.4	0.2
	Os01t0933200-00	0.7	0.8	3.1	1.2	1.0	0.9
	Os10t0537400-00	1.0	1.0	2.4	1.0	1.0	1.0
	Os03t0126700-01	0.6	0.5	2.1	1.0	1.0	2.2
	Os08t0205400-01	1.5	1.7	1.9	0.8	1.2	1.8
	Os03t0111400-01	2.2	3.4	1.9	1.0	1.0	4.4
	Os08t0403300-00	1.0	2.1	1.7	1.0	2.4	**6.4**
	Os10t0506100-01	1.0	1.0	1.7	1.0	1.0	1.2
	Os06t0700700-01	1.3	1.0	1.7	1.7	1.0	1.0
	Os02t0196600-01	1.3	1.2	1.6	1.3	1.4	2.3
	Os12t0421000-01	1.0	1.3	1.6	1.0	1.0	2.0
	Os01t0595201-00	2.4	2.6	1.5	1.0	1.0	1.0
	Os02t0172600-00	1.5	1.4	1.5	1.0	1.3	1.5
	Os04t0556000-01	2.8	2.3	1.3	1.0	1.0	3.7
	Os10t0532300-01	0.8	0.8	1.3	1.0	1.5	2.3
	Os04t0581800-01	1.0	1.0	1.0	1.0	2.5	1.0
	Os01t0249700-00	1.0	1.0	1.0	1.0	1.0	1.0
	Os01t0758000-00	1.0	1.0	1.0	1.0	1.0	1.0
	Os02t0819000-00	1.0	1.0	1.0	1.0	1.0	1.0
	Os03t0156600-01	1.0	1.0	1.0	1.0	1.0	1.0
	Os03t0383900-01	1.0	1.0	1.0	1.0	1.0	1.0
	Os05t0368600-01	1.0	0.5	1.0	1.0	1.0	1.0
	Os01t0309800-01	1.0	1.0	1.0	1.3	0.9	0.4
	Os03t0388100-02	1.0	1.0	1.0	0.5	0.4	0.4
	Os03t0388100-01	1.0	1.0	1.0	0.5	0.5	0.4
	Os03t0751600-02	1.0	1.0	1.0	1.0	1.0	0.3
	Os03t0751600-01	1.0	1.0	1.0	1.0	1.0	0.3
	Os01t0507700-01	1.0	0.7	0.8	1.0	1.4	1.0
	Os07t0298900-01	1.0	0.5	0.7	1.0	1.0	1.0
	Os07t0623200-02	1.0	1.0	0.7	1.0	1.0	1.1

	Os07t0623200-03	1.0	1.0	0.7	1.0	1.0	1.0
	Os03t0178100-00	1.0	1.0	0.7	1.1	0.9	**5.2**
	Os07t0232900-00	0.7	0.5	0.6	1.0	1.0	0.6
	Os07t0623200-01	1.0	1.0	0.6	1.0	1.0	1.0
	Os05t0534500-01	1.0	1.0	0.6	1.0	1.0	1.0
	Os01t0927300-01	1.0	1.0	0.6	0.5	1.0	0.6
	Os01t0192500-00	1.0	1.0	0.5	1.0	1.0	1.0
	Os07t0671400-01	1.0	1.0	0.5	1.0	1.0	0.6
	Os04t0573200-01	1.0	1.0	0.4	1.0	1.0	1.1
	Os04t0573200-02	1.0	1.0	0.4	1.0	1.0	1.0
	Os04t0590100-00	2.4	1.0	0.4	1.0	1.0	1.0
	Os03t0120400-01	1.0	0.7	0.4	1.0	0.5	0.1
	Os08t0512200-00	1.0	1.0	0.3	1.0	1.0	1.0
	Os03t0819400-01	1.0	1.0	0.3	1.0	1.0	0.4
	Os02t0510600-01	1.0	1.0	0.3	0.5	0.6	1.2
	Os01t0719600-01	1.0	0.7	0.3	1.0	1.0	0.0
	Os01t0678800-01	1.6	1.7	0.3	1.0	1.0	1.0
	Os04t0390100-01	1.0	1.5	0.2	1.0	1.0	4.6
	Os12t0144600-01	1.0	1.0	0.2	1.0	1.0	0.7
	Os11t0147500-01	1.0	1.4	0.2	1.0	1.0	0.6
	Os11t0147500-02	1.0	1.0	0.2	1.0	1.0	0.6
	Os01t0125600-01	0.7	0.8	0.1	1.0	1.0	0.7
	Os03t0861400-00	1.0	1.0	0.1	1.0	1.0	1.0
	Os08t0405700-01	0.6	0.4	0.1	1.0	1.0	1.0
	Os10t0344000-01	**7.3**	3.9	**12.2**	1.0	1.0	**13.6**
	Os03t0188100-01	1.8	1.7	**9.8**	2.1	2.8	**14.5**
	Os10t0345100-01	0.7	0.7	4.5	1.0	1.0	**6.7**
	Os06t0494400-01	1.6	2.3	4.9	1.3	1.4	2.2
	Os10t0344500-00	2.0	1.0	3.9	1.0	1.5	1.2
	Os03t0571900-01	1.0	2.3	2.6	1.0	1.6	1.7
	Os04t0571600-01	0.5	0.3	2.5	1.0	0.5	0.2
	Os04t0373400-01	1.5	0.7	2.1	1.0	1.0	4.0
	Os01t0504500-01	1.6	2.0	1.9	1.4	1.6	4.2
	Os12t0125800-00	1.0	1.0	1.9	1.0	1.0	1.0
	Os06t0495100-00	1.0	1.0	1.6	1.0	1.0	2.4
	Os10t0190900-01	1.0	1.0	1.6	1.0	1.0	2.1
	Os11t0126100-01	1.0	1.0	1.6	1.0	1.0	0.4
	Os03t0626700-01	1.0	1.0	1.4	1.0	1.0	1.0
	Os07t0502200-01	1.0	2.0	1.3	1.0	1.1	**6.2**
	Os03t0572900-01	1.0	1.3	1.0	1.0	1.0	**7.4**
	Os08t0480000-01	0.6	0.5	1.0	1.0	0.7	1.3
	Os01t0766000-00	1.0	1.0	1.0	1.0	1.0	1.0
	Os02t0821600-00	1.0	1.0	1.0	1.0	1.0	1.0
	Os06t0558300-00	1.0	1.0	1.0	1.0	1.0	1.0
	Os12t0552600-00	1.0	1.0	1.0	1.0	1.0	1.0
	Os05t0554000-02	0.8	0.8	1.0	1.0	1.0	1.0
	Os03t0858800-01	0.4	0.3	1.0	1.8	2.4	1.0
	Os05t0554000-01	0.8	0.7	1.0	1.0	1.0	0.7
	Os11t0129200-00	1.0	1.0	1.0	1.0	1.0	0.3
	Os03t0229500-00	0.4	0.3	1.0	1.0	0.5	0.2
MatE	*Os03t0571700-01*	1.0	1.0	1.0	1.6	1.7	0.1
	Os01t0504500-02	1.5	1.9	0.9	1.2	1.3	**5.4**
	Os01t0684900-01	1.0	1.3	0.8	1.0	0.7	1.6
	Os11t0129000-00	1.0	1.7	0.8	1.0	1.0	0.3
	Os12t0126000-01	2.1	2.6	0.7	1.4	1.4	1.0
	Os09t0524300-00	1.0	1.0	0.7	1.0	1.0	0.8
	Os03t0216700-01	0.7	0.4	0.7	1.7	1.4	1.0
	Os02t0676400-00	0.5	1.0	0.7	1.0	1.0	1.0
	Os06t0707100-01	1.0	1.0	0.7	1.0	1.3	1.2
	Os12t0106600-01	1.0	1.0	0.7	1.0	1.0	0.5
	Os02t0122200-00	1.0	1.0	0.6	1.0	1.2	2.3
	Os08t0545900-00	1.0	1.0	0.6	1.0	1.0	1.0
	Os07t0516600-01	0.5	1.0	0.6	1.0	1.0	0.3
	Os12t0615700-01	1.0	1.0	0.6	1.0	1.0	0.7

	Os03t0839200-01	1.0	1.3	0.6	1.0	0.6	0.2
	Os08t0550200-01	1.0	1.0	0.6	1.0	1.0	0.8
	Os09t0468000-01	1.0	1.0	0.6	1.0	1.0	0.8
	Os08t0562800-01	0.6	0.6	0.5	1.0	1.0	0.5
	Os09t0548300-01	1.0	0.8	0.4	1.0	1.3	0.5
	Os01t0919100-00	1.0	1.0	0.4	1.0	1.0	0.8
	Os08t0562800-02	0.6	0.6	0.4	1.0	0.8	0.3
	Os10t0195000-01	1.0	0.2	0.4	1.0	1.0	0.6
	Os10t0206800-01	0.4	0.4	0.3	1.0	1.1	0.5
	Os10t0206800-02	0.4	0.4	0.3	1.0	1.0	0.5
	Os07t0108200-00	1.0	1.9	0.3	1.0	1.0	1.0
	Os03t0570800-01	1.0	0.6	0.2	1.5	1.6	0.7
	Os06t0495500-01	1.0	1.0	0.2	1.0	1.0	1.0
	Os10t0344900-01	0.4	0.5	0.2	1.0	1.0	1.0
Nramp	*Os07t0258400-02*	2.3	1.5	**5.0**	1.0	1.0	1.5
	Os07t0258400-01	2.3	1.6	**5.0**	1.0	1.0	1.7
	Os03t0208500-01	1.3	1.3	2.1	1.0	1.0	1.6
	Os12t0581600-01	1.0	1.0	1.6	0.6	0.7	0.8
	Os06t0676000-01	1.2	1.3	1.6	1.0	1.0	1.0
	Os01t0503400-05	2.5	2.1	1.5	1.0	1.0	0.8
	Os01t0503400-04	2.1	1.9	1.4	1.0	1.0	1.3
	Os01t0503400-03	2.2	1.7	1.3	0.8	0.8	0.5
	Os03t0607400-01	1.0	1.0	1.0	1.0	1.0	1.2
	Os01t0733001-00	0.3	0.3	1.0	1.0	1.0	1.0
	Os03t0606600-00	1.0	1.0	1.0	1.0	1.0	0.7
	Os03t0700800-02	1.0	1.0	0.6	1.5	1.6	1.6
	Os03t0700800-01	1.0	1.0	0.6	1.3	1.4	0.9
	Os07t0257200-01	1.0	0.5	0.3	1.9	1.8	0.4
	Os02t0131800-01	0.5	0.2	0.0	1.0	1.0	0.7
PDR_assoc	*Os01t0342750-01*	1.0	1.6	**5.8**	1.0	1.0	2.5
	Os01t0609900-02	3.2	4.0	2.1	0.8	0.7	**32.0**
	Os01t0609300-01	4.3	**6.8**	2.0	1.0	1.0	**32.0**
	Os08t0384500-01	3.0	3.8	1.3	0.7	0.7	**5.2**
	Os01t0516900-00	1.0	1.0	1.0	1.0	1.0	1.0
	Os09t0332300-00	1.0	1.0	1.0	1.0	1.0	1.0
	Os09t0333000-00	1.0	1.0	1.0	1.0	1.0	1.0
	Os06t0554800-01	2.0	2.4	0.8	1.1	0.9	0.8
	Os01t0609200-00	1.0	2.5	0.7	1.0	1.0	1.1
	Os01t0724500-01	1.0	1.0	0.6	1.0	1.0	1.0
	Os12t0512700-01	1.0	0.7	0.5	1.0	1.0	1.0
	Os01t0609000-00	1.8	1.0	0.4	1.0	1.0	0.9
Zip	*Os03t0411800-01*	2.3	2.3	**10.6**	1.0	1.0	**14.6**
	Os04t0613000-01	1.0	1.0	2.0	1.0	1.0	1.0
	Os03t0667500-01	**6.3**	**6.7**	2.0	1.0	1.0	0.7
	Os01t0972200-00	1.0	1.0	1.7	1.0	1.0	2.1
	Os07t0232800-00	1.0	2.5	1.5	1.0	1.4	1.3
	Os03t0667300-00	2.7	1.0	1.4	1.0	1.0	1.0
	Os08t0207500-01	0.8	1.4	1.3	1.0	1.0	0.1
	Os05t0164800-01	1.0	1.0	1.0	1.0	1.0	1.0
	Os05t0164800-02	1.0	1.0	1.0	1.0	1.0	1.0
	Os05t0198400-01	0.5	0.6	0.8	1.0	1.0	0.3
	Os05t0316100-01	1.0	0.8	0.7	1.0	1.2	1.0
	Os05t0316100-02	1.0	0.8	0.7	1.0	1.2	0.8
	Os08t0467400-01	0.8	1.0	0.6	1.0	1.3	1.0
	Os08t0467400-02	0.8	1.0	0.6	1.0	1.4	1.2
	Os08t0467400-03	0.8	1.0	0.6	1.0	1.4	1.2
	Os02t0196000-01	1.0	0.7	0.5	1.0	1.0	0.8
	Os06t0566300-00	1.0	1.8	0.3	1.0	1.0	0.5
	Os05t0472700-01	0.5	0.7	0.3	1.0	1.0	0.4
	Os05t0472400-00	0.3	0.3	0.2	1.0	1.0	1.0

Gene expression of metal ion transporters containing Pfam domains [PF01554 (MatE), PF08370 (PDR_assoc), PF01545 (Cation_efflux), PF02535 (Zip), PF00403 (HMA), PF01566 (Nramp)] is shown. Bold characters show fold changes greater than 5 in one or more tissues/treatments.
